# Characterisation of complete high- and low-risk human papillomavirus genomes isolated from cervical specimens in southern Brazil

**DOI:** 10.1590/0074-02760170121

**Published:** 2017-10

**Authors:** Gisele R de Oliveira, Juliana D Siqueira, Fabiana Finger-Jardim, Valdimara C Vieira, Ronald L Silva, Carla V Gonçalves, Esmeralda A Soares, Ana Maria Barral de Martinez, Marcelo A Soares

**Affiliations:** 1 Universidade Federal do Rio Grande, Escola de Medicina, Laboratório de Biologia Molecular, Rio Grande, RS, Brasil; 2 Instituto Nacional de Câncer, Programa de Oncovirologia, Rio de Janeiro, RJ, Brasil; 3 Universidade Federal do Rio Grande, Escola de Medicina, Rio Grande, RS, Brasil; 4 Universidade Federal do Rio Grande, Escola de Medicina, Centro de Obstetrícia e Ginecologia, Rio Grande, RS, Brasil

**Keywords:** human papillomavirus, lineage, complete genome, southern Brazil

## Abstract

The classification of human papillomavirus (HPV) intratypic lineages by complete genome sequencing is a determinant in understanding biological differences in association with this disease. In this work, we have characterised complete HPV genomes from southern Brazil. Fifteen cervicovaginal Pap smear negative samples previously categorised as HPV-positive were sequenced using ultradeep sequencing, and 18 complete genomes from 13 different HPV types were assembled. Phylogenetic and genetic distance analyses were performed to classify the HPV genomes into lineages and sublineages. This is the first report describing the distribution of HPV intratype lineages of high and low oncogenic risk in asymptomatic women from southern Brazil.

Human papillomavirus (HPV) is the most prevalent sexually transmitted infection worldwide and is a major factor in the development of cervical cancer (zur Hausen 1976). With over 200 different human types already identified, HPVs are classified according to their carcinogenic potential into low- and high-risk types (Muñoz et al. 2003, de Villiers et al. 2004).

Distinct HPV types differ by more than 10% in their L1 sequences, whereas the classification of intratype lineages and sublineages requires complete genome information. Nucleotide distances between 1 and 10% distinguish intratype lineages, whereas differences between 0.5 and 1% define distinct sublineages (Burk et al. 2013).

Biological differences have been proposed for HPV lineages and sublineages (Burk et al. 2013). Studies on the clinical relevance of HPV lineages have focused mainly on HPV16 and 18, associated with differential risks for cervical cancer (Chen et al. 2015, Mirabello et al. 2016). Recently, next-generation sequencing (NGS) technologies have emerged to examine viral diversity in clinical specimens (Radford et al. 2012). These techniques have enabled the identification of HPV genotypes that have not been identified by conventional molecular techniques (Meiring et al. 2012). The purpose of the present study was to characterise full-length HPV genomes and their intratype classification from cervicovaginal samples of women with negative Pap tests living in Rio Grande city, southern Brazil, by NGS.

Fifteen samples of cervicovaginal smears originated from HPV-positive women previously typed by Oliveira et al. (2013) were studied herein. Samples originated from regularly screened women who were found to be Pap smear-negative and were collected during routine gynaecological care at the Gynaecology and Obstetrics Clinic at the Hospital Universitário Dr Miguel Riet Corrêa Jr, in Rio Grande, RS, Brazil. This study has been approved by the Universidade Federal do Rio Grande (FURG) Health Research Ethics Committee (CEPAS No. 013/2011). HPV infection by types 6, 16, 18, 31, 33, 35, 58, 67, 68, 82 and 83 was previously determined in samples described in Oliveira et al. (2013) using standard Sanger sequencing.

HPV DNA was first enriched by rolling circle amplification (RCA) using the Illustra TempliPhi DNA Amplification kit (GE Healthcare Life Sciences, New Jersey, USA). Libraries were prepared for each sample using a Nextera^®^ XT DNA Sample Preparation kit (Illumina Inc., San Diego, USA) and tagmentation and polymerase chain reaction (PCR) according to the protocol established by the manufacturer. Libraries were purified according to the manufacturer’s protocol and quantified by real-time PCR in an ECO System (Illumina Inc.) using the Kapa Library Quantification kit (Kapa Biosystems, Wilmington, USA). Pooled libraries were subjected to clustering in a cluster C-bot station and sequenced in an Illumina^®^ HiSeq 2500 system. After sequencing, reads were filtered in the Sickle-Master programme (available from: https://github.com/najoshi/sickle) to select reads with a quality ≥ 28 in the Phred scale and with a length ≥ 20 bp. Reads were evaluated with FastQC software (Bioinformatics Babraham, Cambridge, UK) for their amount and mean quality.

BWA (Li & Durbin 2009) was used to assemble the HPV genomes in which reads were mapped to HPV reference sequences. One hundred and sixty-seven different HPV type reference sequences were retrieved from the HPV PAVE database (http://pave.niaid.nih.gov/) and used in the assemblies. The consensus sequences from each assembly (for each sample) were extracted and the sequences with size ≥ 97.5% of the reference genome were considered complete or near-complete genomes. All HPV complete genome sequences generated herein have been deposited in the GenBank Sequence Database and have been assigned the accession numbers KX514416-KX514433.

An alignment of each HPV type was prepared with the complete genomes generated and representative genomes of different lineages and/or sublineages of HPV as defined by Burk et al. (2013) in ClustalW2 (Larkin et al. 2007). The difference between the genomes generated and the references was calculated by the *p*-distance method in MEGA5 (Tamura et al. 2011) and used for classification of sequences into HPV lineages and sublineages.

Over 2 million reads were obtained for each sample after trimming for quality, and an average of 4.16% of reads were mapped to HPV reference sequences of 167 different types. Eighteen complete or near-complete HPV genomes were assembled with approximately 8.8 kb (97.5% to 100% of genome) in 12 out of the 15 samples analysed. The remaining three samples did not provide enough information that enabled HPV complete genome assembly.

The 18 complete genomes, spanning 13 different HPV types of the alpha genus (HPV6 (n = 3), HPV18 (n = 1), HPV31 (n = 2), HPV35 (n = 1), HPV39 (n = 1), HPV51 (n = 1), HPV56 (n = 1), HPV58 (n = 1), HPV61 (n = 1), HPV67 (n = 1), HPV68 (n = 2), HPV82 (n = 2) and HPV85 (n = 1)), were aligned with reference sequences from different lineages and sublineages of each HPV type (Burk et al. 2013). Newly assembled complete HPV genomes were classified into lineages and sublineages considering a p-distance < 0.5% compared to the respective lineage/sublineage reference sequence (Table). Double or multiple infections were detected by NGS in 53% (8/15) of the previously typed samples, and 14 infections of different HPV types were found in some samples that had not been previously detected by Sanger sequencing as well (Table).

**TABLE:** Distribution of human papillomavirus (HPV) types, multiple infections, percent nucleotide distance of each HPV complete genome and identified lineages and sublineages by next-generation sequencing infecting women followed-up at University Hospital of the Federal University of Rio Grande

Sample	HPV type by Sanger sequencing*	HPV type(s) by NGS	Complete genomes assembled	Distance (%)	Lineage(s)/ sublineage(s) assigned
01	6	6	6	0.1	B3
02	6	6	6	0.1	B1
03	6	6	6	0.2	A
04	16	16, 31	---	---	---
05	18	18	18	0.1	A3
06	31	31, 68	31 68	0.3 0.3	C2 C1
07	33	68	68	0.2	C1
08	35	35, 39	35 39	0.3 0.1	A1 A1
09	58	51, 58	51	0.2	A1
10	58	58	58	0.1	A2
11	67	31, 67, 85	67 85 31	0.1 0.1 0.3	A2 A B2
12	82	56, 61, 82	56 61 82	0.2 0.1 0.1	A2 A1 A2
13	82	82	82	0.1	A2
14	83	31, 82, 83,	---	---	---
15	83	6, 74, 82, 83	---	---	---

*: HPV previously typed by Oliveira et al. (2013); NGS: next-generation sequencing.

This report is the first describing the distribution of HPV lineages/sublineages of high and low oncogenic risk in asymptomatic (Pap smear-negative) women from southern Brazil. We showed herein that the use of NGS enabled the detection of co-infections with multiple HPV genotypes due to its greater sensitivity when compared to Sanger sequencing, as shown previously by our group and others (da Fonseca et al. 2016, Siqueira et al. 2016).

Interestingly, in one of the samples (07) the HPV type identified by Sanger sequencing was not detected by NGS, even though the latter is more sensitive. One possible explanation for this observation may be related to the use of the rolling circle amplification (RCA) enrichment during the preparation of samples for NGS, a technique that enriches episomal (unintegrated) HPV genomes. Thus, we hypothesize that HPV33, which was previously detected by Sanger sequencing in sample 07, is likely integrated, and its detection was disfavoured by the RCA protocol, while HPV68 may be episomal, and may have been enriched during RCA and hence detected. This may be considered an important limitation to our study and to the use of NGS for HPV typing. While amplicon-based NGS approaches could circumvent this bias (Lavezzo et al. 2016), they do not allow HPV full-length determination, a requirement for lineage/sublineage classification.

In this study, the lineages identified that HPV6, the aetiological agent of anogenital warts and laryngeal papillomas, belonged to the A lineage and B1 and B3 sublineages. These results are in agreement with the multicentric study by Jelen et al. (2014), who identified the same HPV6 lineages and sublineages associated with anogenital infections in Argentina, a country which borders southern Brazil.

With respect to HPVs with high oncogenic potential, HPV18 is the second most common type associated with cervical cancer. Our study has identified an European sublineage of HPV-18 (A3) in a sample of a woman with no cervical intraepithelial lesions. Previous work has also identified European HPV-18 lineages as predominant among asymptomatic women in southeastern Brazil (Villa et al. 2000). A high frequency (70%) of HPV-18 lineage A was found in women with invasive cervical cancer in a recent study in southeastern Brazil (Vidal et al. 2016), which may underscore the oncogenic potential of the infection found herein.

HPV58 is the second highest oncogenic risk type in prevalence in the city of Rio Grande, Brazil (Oliveira et al. 2013). In this study, the lineage of HPV58 identified in asymptomatic women belonged to the A2 sublineage. A study performed in another region of Brazil (Siqueira et al. 2016), also identified the same strain in asymptomatic women. However, Mejía et al. (2016) identified in Ecuador the same sublineage of HPV58 in women with cervical intraepithelial lesions, which may suggest that infection by HPV58 A2 sublineage can be a risk factor for the development of cervical intraepithelial lesions. Despite HPV intratype diversity being previously appreciated in Brazil for types 31, 35 and 58 (also characterised herein) (Calleja-Macias et al. 2005, Raiol et al. 2009), the lack of correspondence to the recent lineage/sublineage classification as proposed by Burk et al. (2013) did not allow a direct comparison of diversity data for those types.

HPV is the most prevalent sexually transmitted infection worldwide and a major factor for the development of cervical cancer. Infection by multiple HPV types or lineages can be pivotal to the virus’s oncogenic potential in infected women. Next-generation sequencing becomes a powerful research tool to identify co-infections by different HPV types and/or lineages worldwide.
